# Comprehensive automation of the solid phase extraction gas chromatographic mass spectrometric analysis (SPE-GC/MS) of opioids, cocaine, and metabolites from serum and other matrices

**DOI:** 10.1007/s00216-014-7815-7

**Published:** 2014-05-02

**Authors:** Oliver Lerch, Oliver Temme, Thomas Daldrup

**Affiliations:** 1 GERSTEL GmbH & Co. KG, Eberhard-Gerstel-Platz 1, 45473 Muelheim, Germany; 2 Department of Forensic Toxicology, Institute of Legal Medicine, University Hospital Duesseldorf, Moorenstrasse 5, 40225 Duesseldorf, Germany

**Keywords:** Opiates, Opioids, Cocaine, Solid phase extraction (SPE), Automation, GC/MS

## Abstract

The analysis of opioids, cocaine, and metabolites from blood serum is a routine task in forensic laboratories. Commonly, the employed methods include many manual or partly automated steps like protein precipitation, dilution, solid phase extraction, evaporation, and derivatization preceding a gas chromatography (GC)/mass spectrometry (MS) or liquid chromatography (LC)/MS analysis. In this study, a comprehensively automated method was developed from a validated, partly automated routine method. This was possible by replicating method parameters on the automated system. Only marginal optimization of parameters was necessary. The automation relying on an x-y-z robot after manual protein precipitation includes the solid phase extraction, evaporation of the eluate, derivatization (silylation with *N*-methyl-*N*-trimethylsilyltrifluoroacetamide, MSTFA), and injection into a GC/MS. A quantitative analysis of almost 170 authentic serum samples and more than 50 authentic samples of other matrices like urine, different tissues, and heart blood on cocaine, benzoylecgonine, methadone, morphine, codeine, 6-monoacetylmorphine, dihydrocodeine, and 7-aminoflunitrazepam was conducted with both methods proving that the analytical results are equivalent even near the limits of quantification (low ng/ml range). To our best knowledge, this application is the first one reported in the literature employing this sample preparation system.

## Introduction

Analysis of drugs and metabolites in biological fluids or tissues usually requires sample preparation for cleanup and enrichment. In recent years, in conjunction with very sensitive and selective mass spectrometers, protein precipitation alone [1] or “dilute and shoot” methods [2, 3] were also used whereat these methods may suffer from sample-dependent matrix effects able to compromise the accuracy of the results. Although the number of LC-MS/MS methods is rapidly increasing [4–7], gas chromatography (GC)-mass spectrometry (MS)/(MS) is still the standard routine analysis instrument in many forensic laboratories [4, 8–10].

A variety of sample preparation techniques like liquid–liquid extraction (LLE) [11], supported liquid extraction (SLE) [12–15], static headspace extraction (HS), and solid phase micro extraction (SPME) [16] is used.

Solid phase extraction (SPE) is one of the most popular extraction techniques for toxicological analyses of biological fluids and tissues [4, 12, 17]. Usually, polypropylene cartridges with a fixed sorbent bed (e.g., mixed mode cation exchange cartridges) are applied.

Numerous systems for automating SPE are commercially available [18]. In many forensic laboratories, benchtop systems like the ASPEC from Gilson [5, 6, 19] or the RapidTrace from Biotage [20–22] which employ standard SPE cartridges and mimic the manual workflow are used. Complete automation of sample preparation and analysis is possible with online SPE systems where the SPE cartridge is integrated into an LC flow path. Systems with nonautomatically exchangeable [23, 24] and automatically exchangeable [25–27] cartridges are available. Online coupling of SPE to GC is less common and more complex [12] but an important step to reduce manual work and handling errors.

Numerous methods for the analysis of opioids, cocaine, and metabolites in different matrices were published. The compounds were analyzed from different matrices like urine [9, 28, 29], whole blood, serum, plasma [5, 9, 7], saliva [6], hair [8], or post-mortem samples [17]. In this study, a completely automated SPE-GC/MS method was developed from a validated, partly automated (SPE) analysis method. Automation is performed by different modules attached to an x-y-z robot. This allows mimicking the manual workflow of sample dilution, SPE, evaporation, derivatization, and sample injection. Almost 170 authentic serum samples and more than 50 authentic samples of other matrices were analyzed in parallel with both methods. Equivalence of results therefore, the validity of the method and the suitability of the automated system for routine forensic analysis, should be proven by this study design.

## Materials and methods

### Solvents, reagents, standards, and materials

All analytes and deuterated analogs were certified standards. 7-Aminoflunitrazepam (1 mg/ml in acetonitrile) and dihydrocodeine (1 mg/ml in methanol) were purchased from Lipomed (Arlesheim, Switzerland). 7-Aminoflunitrazepam-d_7_, 6-monoacetylmorphine, 6-monoacetylmorphine-d_3_ (each 0.1 mg/ml in acetonitrile), methadone, morphine, morphine-d_3_, codeine, codeine-d_3_, dihydrocodeine-d_6_ (each 0.1 mg/ml in methanol), methadone-d_9_, benzoylecgonine, benzoylecgonine-d_3_ (each 1 mg/ml in methanol), cocaine, and cocaine-d_3_ (each 1 mg/ml in acetonitrile) were purchased from LGC Promochem (Wesel, Germany). For calibration and solvent quality controls, multi-compound solutions and one multi-compound internal standard solution containing deuterated analogs of every analyte were prepared in methanol. Each 20 μl of the internal standard solution was added to samples, calibration samples, and quality controls. Blood, urine, and tissue samples were taken from authentic forensic cases of the Institute of Legal Medicine (Duesseldorf, Germany). Internal controls consisting of drug-negative serum (given by the Center of Haemostaseology, University Hospital Duesseldorf, Germany) and the above-mentioned standards were prepared by the Institute of Legal Medicine. The external controls were certified testing materials (lyophilized serum “Medidrug BTMF 2 S-plus” purchased from Medichem, Steinenbronn, Germany).

**Fig. 1:**
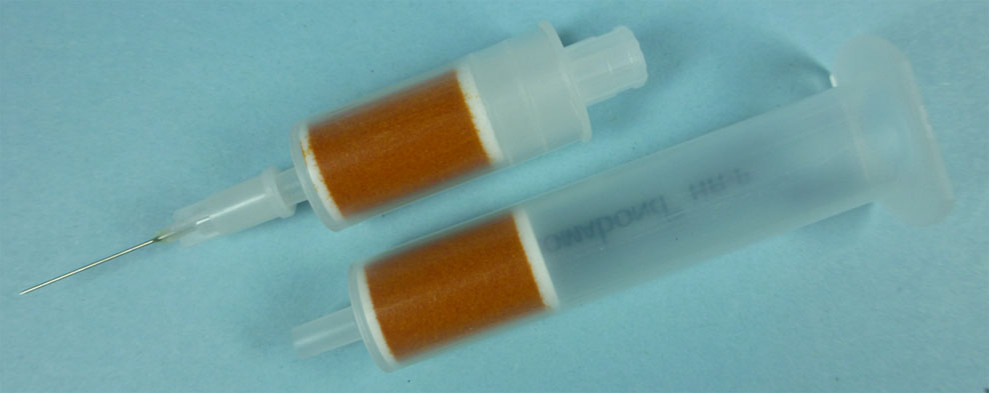
Solid phase extraction (SPE) cartridges. *Top*: Solid phase extraction cartridge equipped for automated SPE. *Bottom*: Standard solid phase extraction cartridge

All solvents and salts were of analytical grade and purchased from VWR (Darmstadt, Germany). *N*-Methyl-*N*-trimethylsilyltrifluoroacetamide (MSTFA) for silylation was from Macherey-Nagel (Dueren, Germany) for the partly automated method and from Sigma-Aldrich (Taufkirchen, Germany) for the automated method. Bond Elut Certify 130 mg, 3 ml format SPE cartridges from Agilent Technologies (Waldbronn, Germany) were used. For automated solid phase extraction, these cartridges were cut at the top, equipped with a transport adapter and a disposable cannula (Fig. 1). Cartridges in such format are commercially available from other vendors like Macherey & Nagel, Sigma-Aldrich, Phenomenex, and Bekolut.

For solid phase extraction, 0.15 M acetic acid, a phosphate buffer of pH 7.9 (4.49 g Na_2_HPO_4_ + 0.2 g KH_2_PO_4_ ad 400 ml H_2_O), and a mixture of dichloromethane/isopropanol/25 % ammonia solution 40/10/1 (*v*/*v*/*v*) for analyte elution were prepared. A mixture of isooctane/MSTFA 19/1 (*v*/*v*) was used for manual derivatization and a mixture of isooctane/pyridine/MSTFA 14/5/1 (*v*/*v*/*v*) for automated derivatization.

Inlet liners and injection vials were deactivated using a solution of 5 % dichlorodimethylsilane in toluene.

### Instrumentation

Measurements with the partly automated, validated analysis method were conducted on a 7890 GC/5975 mass selective detector (MSD) equipped with an HP-5MS column 30 m, *d*_i_ 0.25 mm, and d_f_ 0.25 μm. A 7683B autosampler was used for injection into a hot split/splitless inlet (all Agilent Technologies). For solid phase extraction, a RapidTrace SPE Workstation (Biotage, Uppsala, Sweden) and for evaporation of the eluates a heating block (Medax, Neumuenster, Germany) with ten nitrogen-streamed vial positions (Gebr. Liebisch, Bielefeld, Germany) were used.

**Fig. 2:**
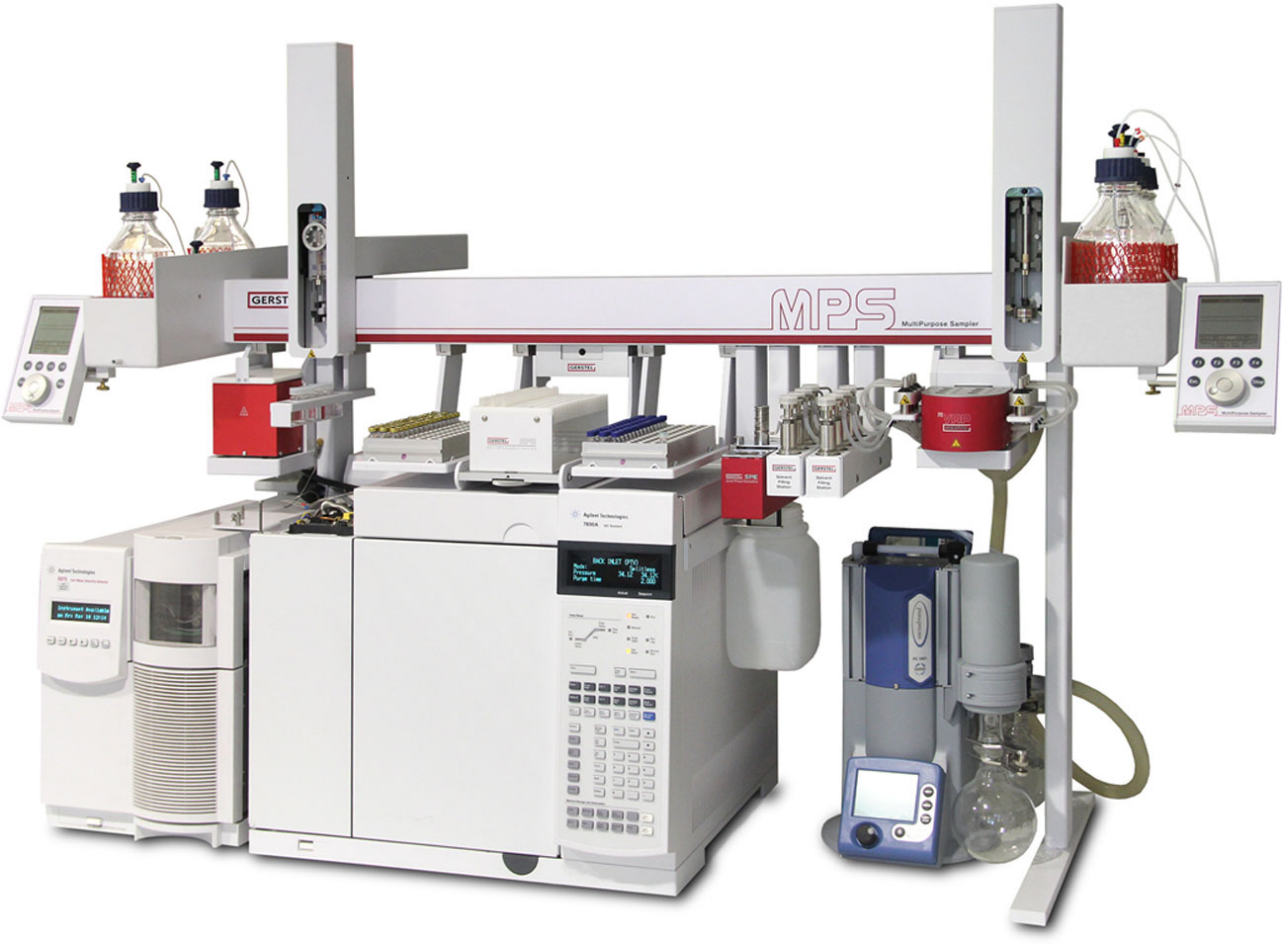
System for automated dilution, solid phase extraction, eluate evaporation, derivatization, injection, and GC/MS analysis. X-y-z dual head robotic autosampler with following modules (from *right to left*): evaporation station, 2× solvent filling station, SPE module, tray for sample vials, tray for SPE cartridges, tray for empty eluate vials, wash station, and agitator

Comprehensive automation of sample preparation—performed in a different laboratory—was based on a MultiPurpose Sampler (MPS) (Fig. 2, GERSTEL, Muelheim, Germany) which is a flexible platform with numerous modules available (e.g., for centrifugation and filtration) so that different sample preparation methods can be automated. It carried two syringes, one 2.5 ml for sample preparation steps and one 10 μl for sample injection into a Programmed Temperature Vaporization (PTV) inlet (Cooled Injection System CIS 4, GERSTEL) coupled to a 6890 GC/5975 MSD (Agilent Technologies). Separation was done on an Rxi-5Sil MS column 30 m, d_i_ 0.25 mm, and d_f_ 0.25 μm from Restek (Bad Homburg, Germany) having very similar retention characteristics as the HP-5MS. Additionally, the autosampler was equipped with a module for SPE, one for evaporation of solvents under controlled vacuum and temperature (multi-position eVAPoration station, mVAP), one for shaking under controlled temperature (Agitator), and one for supplying large volumes of solvents (Solvent Filling Station 2, SFS 2, all GERSTEL). All solvents and samples were delivered by the 2.5-ml syringe which included a gas supply for drying of SPE cartridges.

The RapidTrace SPE Workstation was controlled via RapidTrace SPE Workstation software (Biotage). All MPS sample preparation steps were flexibly combined in and controlled by Maestro software (GERSTEL). The sequential sample preparation steps were automatically overlapped with chromatographic runs and other waiting times so that the GC/MS-system is effectively used. A scheduler window gives a graphical overview over the complete sequence and shows what step is currently executed. ChemStation software (Agilent Technologies) was used to control the GC/MS system and to evaluate the recorded chromatograms.

### Analysis methods

#### Common steps of sample preparation

All liquid samples (urine, blood, serum) and serum controls were handled in the same way: proteins were precipitated by dropwise addition of a mixture of 0.6 ml sample, 0.1 ml water, and 20 μl internal standard solution to a mixture of 1 ml acetonitrile and 0.1 ml isopropanol. After mixing and centrifugation (RCF 17,530×*g*), an aliquot of 0.75 ml of the supernatant was taken for the partly automated analysis method. Another aliquot of 0.75 ml was collected and stored in the freezer at −20 °C for later analysis with the automated system. The storage period for the supernatants ranged from some days to several months.

Tissues (brain and kidney, native and lyophilized) were homogenized with an Ultra Turrax. An aliquot of approximately 0.6 g was weighed and handled like the liquids above, whereas the acetonitrile/isopropanol solution was given to the sample mixture for protein precipitation. The exact weight was noted for calculation of analyte concentrations.

#### Sample preparation: partly automated, validated routine method

A 0.75-ml aliquot of the supernatant of the protein precipitation was diluted with 4.25 ml of phosphate buffer, and the SPE cartridge was conditioned with 2 ml methanol and 2 ml phosphate buffer. After addition of the diluted sample, the cartridge was washed with 2 ml water, 2 ml acetic acid (0.15 M), and 2 ml methanol, shortly dried with nitrogen, and eluted with 2 ml of the mentioned elution solvent.

The eluate was evaporated to dryness in a stream of nitrogen at 60 °C and reconstituted in 0.2 ml of the derivatization solution. After shaking and incubating for 30 min at 90 °C, an aliquot of 2 μl was injected into the GC/MS via a hot inlet at 270 °C.

#### Sample preparation: completely automated method

**Fig. 3:**
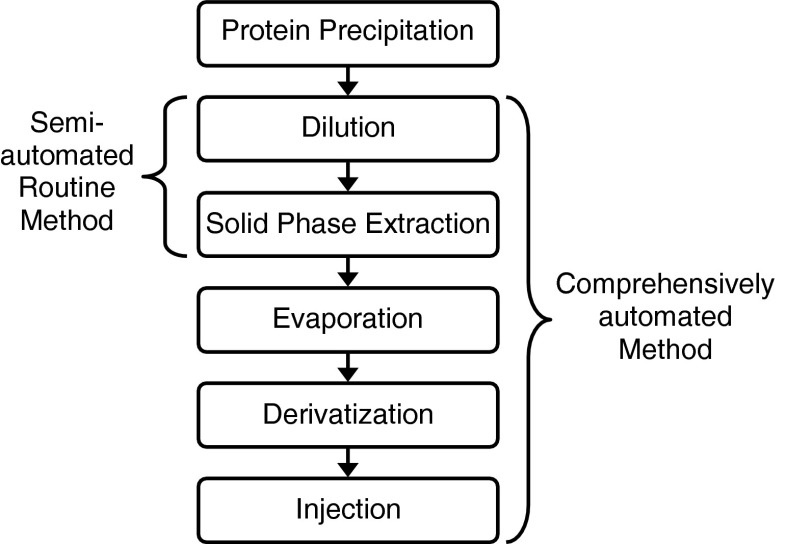
Scheme of the sample preparation workflow indicating automated parts of both methods

The completely automated method was performed analogously to the partly automated method (Fig. 3) described before with some adaptations: Since no adequate vial for taking up the diluted sample (5 ml) was available, a part of the dilution was done in the autosampler syringe before injection into the SPE cartridge. For this, the original sample (supernatant of the protein precipitation) was diluted 1:1 with 0.75 ml of phosphate buffer. Half of this diluted sample (0.75 ml) was aspirated into the syringe followed by 1.75 ml phosphate buffer resulting in the same dilution as in the partly automated method. The syringe content was injected into the SPE cartridge, and the procedure was repeated to transfer the entire sample.

Furthermore, the elution volume was adapted to speed up evaporation and since the preferred elution vial (2 ml format is cheaper than 4 ml format) could not be filled too high for later automated evaporation in the mVAP station. Therefore, an elution profile of the analytes from the SPE cartridge was recorded by fractionated elutions with portions of solvent determining the main analytes containing fractions. The evaporation of the eluate was done in the mVAP station at 70 °C, 300 rpm shaking, and a pump vacuum of 8 kPa. Evaporation time was optimized and finally set to a value of 6 min for reliable evaporation to dryness. In order to speed up the method, the derivatization procedure was modified to the described solvent composition (see “Solvents, reagents, standards, and materials” section) and a reaction time of 5 min.

An aliquot of 2 μl of the derivatized eluate was injected into the CIS 4 (PTV inlet) at 50 °C. The inlet was heated with 12 °C/s to 280 °C held for 5 min.

#### Analysis parameters for GC/MS

Separation was performed on the named columns with constant helium flow of 1 ml/min and the following temperature program: 140 °C (1 min), 120 °C/min, 225 °C (5.29 min), 120 °C/min, 275 °C (5.2 min), and post-run 300 °C (2.5 min) resulting in a cycle time of around 20 min. The MSD was operated in single ion monitoring (SIM) mode recording one quantifier and two qualifier masses (Table 1).

**Table 1:** Quantifier and qualifier ions for analytes and internal standards

	Retention time on Rxi-5MS, min	Quantifier, *m*/*z*	Qualifier, *m*/*z*
Cocaine	7.38	182	303, 198
Cocaine-d_3_	7.36	185	306, 201
Benzoylecgonine	7.76	361	256, 346
Benzoylecgonine-d_3_	7.75	364	259, 349
Methadone	6.57	223	294, 236
Methadone-d_9_	6.49	226^a^, 303^b^	303^a^, 318^b^, 242
Morphine	9.23	429	220, 401
Morphine-d_3_	9.22	432	223, 404
Codeine	8.91	371	234, 343
Codeine-d_3_	8.90	374	237, 346
6-Monoacetylmorphine	9.76	399	340, 400
6-Monoacetylmorphine-d_3_	9.74	402	343, 403
Dihydrocodeine	8.49	373	315, 358
Dihydrocodeine-d_6_	8.46	379	318, 364
7-Aminoflunitrazepam	10.91	326^b^, 355^a^	326^a^, 356^a^, 327^b^, 354^b^
7-Aminoflunitrazepam-d_7_	10.87	362	333, 363

^a^Quantifier/qualifier ion used in partly automated analysis method

^b^Quantifier/qualifier ion used in fully automated analysis method

Deuterated analogs were used for every analyte, and calibration was done via standards in pure methanol. The calibration solution and the methanolic control samples were handled analogously to the eluates. Nine calibration levels were measured in duplicate. Calibrations ranged from 25 to 1,500 ng/ml (methadone), from 50 to 1,500 ng/ml (benzoylecgonine), from 5 to 150 ng/ml (codeine), from 5 to 300 ng/ml (cocaine, dihydrocodeine, morphine), and from 2.5 to 150 ng/ml (7-aminoflunitrazepam, 6-monoacetylmorphine), respectively, and were calculated for 0.6 ml serum samples. Calibration from solvent standards is often used in forensic toxicology and is accepted by the Society of Toxicological and Forensic Chemistry (GTFCh) if equivalence with matrix calibration can be proven.

In between authentic case samples, control samples like negative, low concentration, high concentration, external, and methanolic control were analyzed. According to GTFCh recommendations, a blank injection of pure derivatization solution was done after every sample, quality control, and calibration sample.

## Results and discussion

### Partly automated, validated routine method

The routine method was validated according to the GTFCh guidelines [30]. Validation data are presented in Table 2.

**Table 2:** Validation data of the partly automated routine method

	LOD, ng/ml	LOQ, ng/ml	ULOC, ng/ml	Repeatability^a^, RSD %	Time-different intermediate precision^a^, RSD %	Trueness^a^, bias %	Extraction efficiency^a^, %
Cocaine	1.1	3.5	300	2.4	2.8	+4.6	119
Benzoylecgonine	9	47	1,500	4.1	5.8	+8.9	29
Methadone	4.2	16.7	1,500	3.7	4.6	−1.0	74
Morphine	1.2	4.9	300	6.8	6.8	+13.4	37
Codeine	0.4	2.6	150	6.8	11.3	+2.3	118
6-Monoacetylmorphine	0.3	0.8	150	3.9	5.5	+12.4	78
Dihydrocodeine	0.8	4.2	300	4.1	5.8	+2.8	61
7-Aminoflunitrazepam	0.6	2.5	150	7.0	8.6	+5.5	127

*LOD* limits of detection, *LOQ* limits of quantification, *ULOC* and upper limits of calibration, *RSD* relative standard deviation

^a^Concentrations: 80 ng/ml for benzoylecgonine and methadone; 16 ng/ml for cocaine, morphine, and dihydrocodeine; 4 ng/ml for codeine, 6-monoacetylmorphine, and 7-aminoflunitrazepam

Concentrations of the analytes in the quality controls associated with the sequences were within the allowed tolerance range (according to the GTFCh recommendations of ±30 and ±40 % near the limit of quantification (LOQ), respectively) [30]. The described method is being used for routine analyses in the Institute of Legal Medicine in Duesseldorf for several years. All proficiency tests were passed successfully. The results of the analyzed routine samples were taken as reference for the automated system in order to prove its validity.

### Completely automated method

The partly automated method could be successfully transferred to the completely automated system starting with the dilution of the sample (after protein precipitation) and ending with the injection into the GC/MS. Most parameters could be replicated without need for change. Some parameters were adapted for different reasons discussed in the following.

**Fig. 4:**
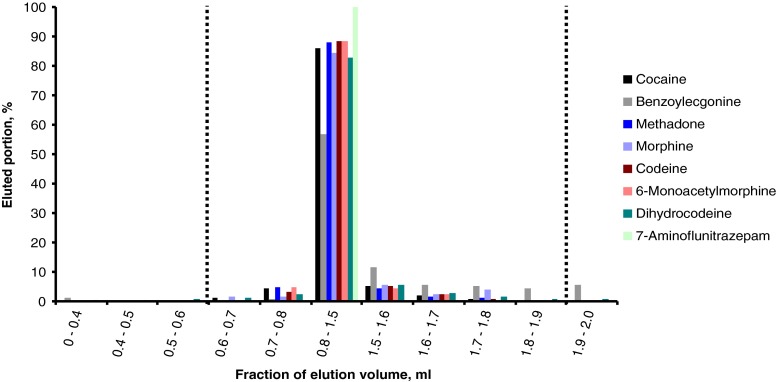
SPE elution profile for all analytes included in this study

The in-syringe dilution worked properly. No carryover for any of the compounds could be detected when extracting blank serum samples after analyte-positive samples. According to the elution profile (Fig. 4), the elution volume was adjusted to 1.9 ml where the first 0.6 ml was discarded and the following 1.3 ml was collected in the elution vial. This volume is adequate for evaporation from a 2-ml vial in the mVAP station. Although the elution solvent consists mainly of the very volatile dichloromethane, the evaporation under controlled vacuum, heating, and shaking was very smooth and repeatable without any boiling retardation.

**Fig. 5:**
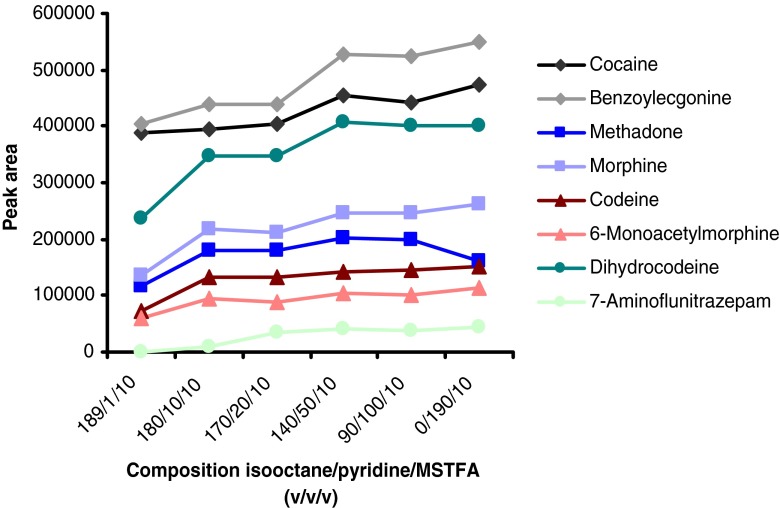
Optimization of derivatization reagent composition for the completely automated method. Pure standards were derivatized with different reagents at 90 °C for 30 min

**Fig. 6:**
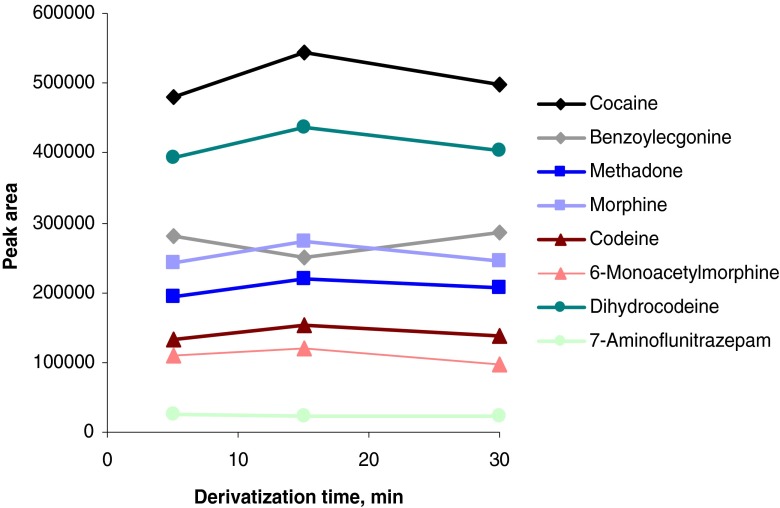
Optimization of derivatization time for the completely automated method. Spiked serum samples were extracted and the eluate was derivatized with isooctane/pyridine/MSTFA 14/5/1 (*v*/*v*/*v*) at 90 °C for different times

The derivatization procedure could be shortened from 30 to 5 min at 90 °C by using a different reagent mixture consisting of isooctane/pyridine/MSTFA 14/5/1 (*v*/*v*/*v*) instead of isooctane/MSTFA 19/1 (*v*/*v*; see Figs. 5 and 6).

For methadone and 7-aminoflunitrazepam, quantifier and qualifier masses are needed to be adapted since coelution occurred. In the case of 7-aminoflunitrazepam, probably siloxanes from vial septa also showing *m*/*z* 355 and 356 coeluted with the analyte. Choosing *m*/*z* 326, 327, and 354 as quantifier/qualifiers could overcome this issue.

All in all, the semiautomated method could readily be transferred to the automated system without major optimization steps.

Almost 170 authentic serum samples and more than 50 authentic samples of other matrices (urine, different tissues, heart blood) were analyzed. All samples were processed properly by the instrument without any failure. By overlapping sample preparation steps with the GC/MS run, a throughput of around 29 samples per day could be achieved which is comparable with the partly automated method. The possible automation of the protein precipitation step was not within the scope of the present work because of the study design (two aliquots of one sample after protein precipitation for parallel analysis). All quantification results for the control samples but one for cocaine and one for benzoylecgonine in an external control sample (2 and 3 % below the allowed tolerance, respectively) were within the allowed tolerance ranges.

### Method comparison

**Fig. 7:**
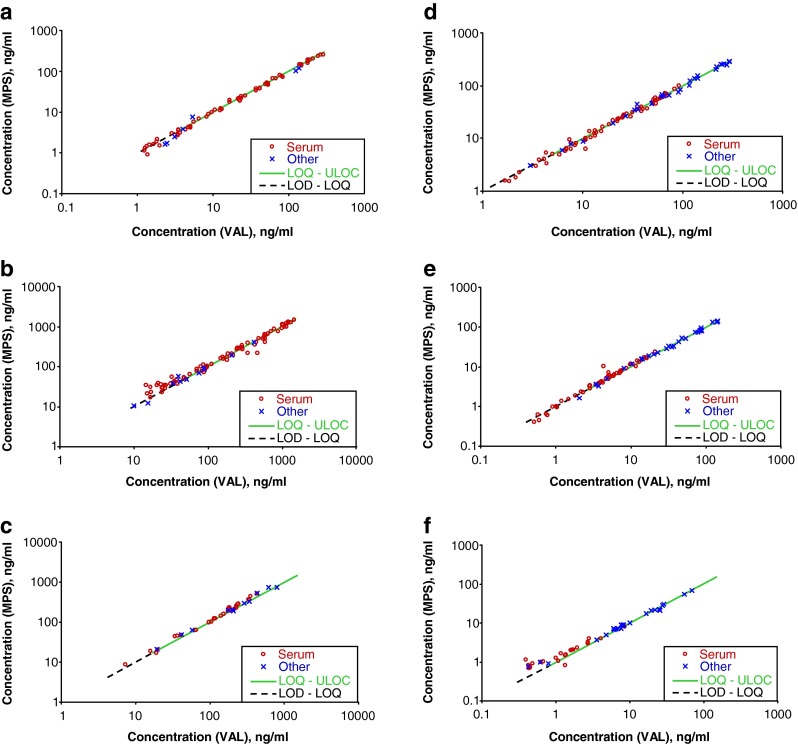
Correlation of measured analyte concentrations in double logarithmic scale. Line with a slope of one (*dashed black* in the range from LOD to LOQ and *solid green* in the range from LOQ to ULOC)—representing the complete equivalence of results—is shown. *VAL* validated partly automated method; *MPS* fully automated method performed on a MultiPurpose Sampler (*MPS*); *ng/ml* nanogram per milliliter or nanogram per gram if tissue is used, respectively; *Other* other matrices than serum—urine, blood, lyophilized kidney tissue, heart blood, lyophilized, and native brain tissue; *LOD* limit of detection; *LOQ* limit of quantification; *ULOC* upper limit of calibration. **a** Cocaine. **b** Benzoylecgonine. **c** Methadone. **d** Morphine. **e** Codeine. **f** 6-Monoacetylmorphine

**Fig. 8:**
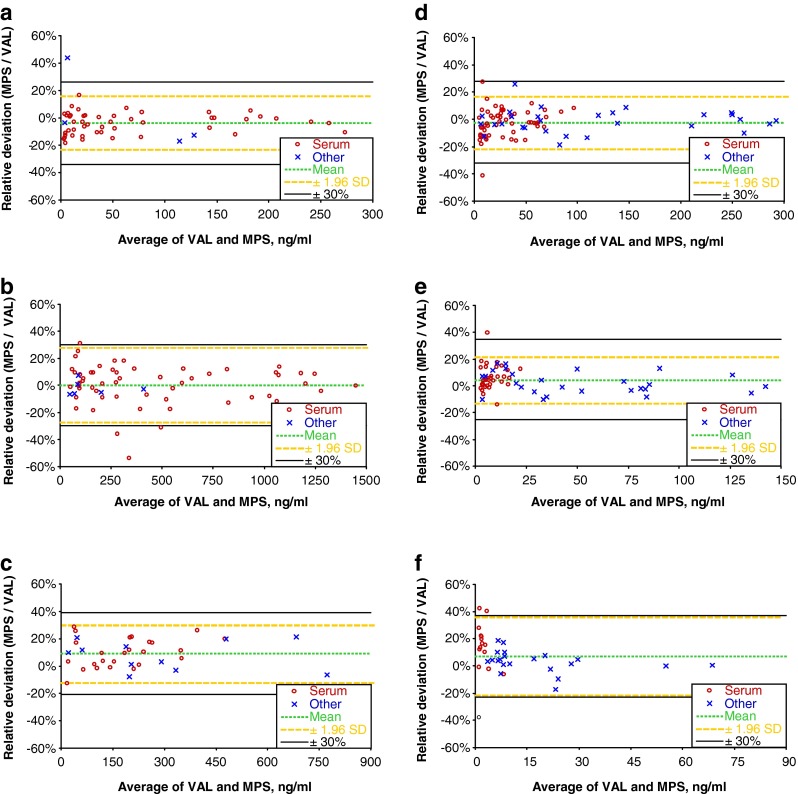
Relative deviations of measured concentrations displayed as Bland-Altman plots. Only analysis values above the limit of quantification are plotted. *VAL* validated partly automated method; *MPS* fully automated method performed on a MultiPurpose Sampler (*MPS*); *ng/ml* nanogram per milliliter or nanogram per gram if tissue is used, respectively; *Other*: other matrices than serum—urine, blood, lyophilized kidney tissue, heart blood, lyophilized, and native brain tissue; *Mean* mean deviation or bias in percent; *SD* standard deviation of percent deviations for each single sample. **a** Cocaine. **b** Benzoylecgonine. **c** Methadone. **d** Morphine. **e** Codeine. **f** 6-Monoacetylmorphine

Results of both methods are equivalent as visible in the double logarithmic line and Bland-Altman plots (Figs. 7 and 8 and Table 3). This is true for serum samples and also for other matrix samples (urine, different tissues, heart blood). Results between the method’s LOQ and the limit of detection (LOD) are included in the line plots (dashed black lines in Fig. 7) as well. Even in this concentration range, the equivalence of both methods is obvious suggesting that LOQ and LOD for both methods are very similar. This indirect validation approach of comparing a large number of authentic samples reveals the accuracy and precision of the comprehensively automated method.

**Table 3:** Statistical data for the automated method in comparison to the validated method inferred from Bland-Altman plots

Analyte	Statistical data from Bland-Altman plots	
	Bias, %	SD, %
Cocaine	−3.6	±10.1
Benzoylecgonine	+0.2	±14.0
Methadone	+9.1	±10.7
Morphine	−2.3	±9.9
Codeine	+6.5	±18.0
6-Monoacetylmorphine	+7.2	±14.7
Dihydrocodeine^a^	−	−
7-Aminoflunitrazepam^a^	−	−

*Bias* mean deviation between both methods in percent, *SD* standard deviation of percent deviations for each single sample

^a^No Bland-Altman plot since only few positive samples were available

The analyses were run in different laboratories by different personnel at different times revealing the ruggedness of the instrumentation and both methods. Since only a couple of samples were positive for dihydrocodeine and 7-aminoflunitrazepam, these results are not plotted. Samples and quality control samples were also in good concordance for these compounds.

LOQs are in the range of other published methods, e.g., Kjaergaard Bjork et al. achieved LOQs between 2.5 and 10 ng/mL in whole blood analysis for the compounds mentioned in this work [5] and Ferreiros Bouzas et al. between 0.5 and 2.8 ng/ml from serum analyzed by LC-MS/MS [7]. According to Jones et al. [9] and Namera et al. [31], LOQs for different opiates in whole blood or serum, respectively, were 5 ng/ml.

## Conclusions

A unique system which mimics complex sample preparation workflows (dilution, SPE, evaporation, derivatization, injection) was employed in this study. Whereas other robotic systems enable the standalone automation of only one sample preparation step, e.g., SPE [5, 6, 19–22] or evaporation, applications employing such comprehensive systems were rarely reported before in scientific literature. An application utilizing this specific system (autosampler plus individual modules) is even reported for the first time. In our opinion, comprehensive automation without manual intervention after putting the sample onto the autosampler offers full benefit to the user.

These are the main achievements and benefits:

- Comprehensive automation of a validated, partly automated analysis method for opioids, cocaine, and metabolites from blood serum and other matrices
- Analysis results of both methods are equivalent (more than 220 authentic samples) on the basis of GTFCh recommendations
- The automated method saves manual work and reduces the risk of human errors. This makes analysis quality projectable and more independent of the users’ experience
- Suitable throughput of samples adapted to the GC/MS analysis time despite the mainly serial workflow
- The automated method proved to be rugged and suitable for routine analysis in forensic laboratories
- The automated analysis system is highly flexible. Therefore, it can be employed for easy automation of other validated GC or LC analysis methods or for standalone automation of sample preparation. As proven by this work, method transfer is readily possible mainly by replicating method parameters on the automated system
